# Mechanisms of enhancer‐driven oncogene activation

**DOI:** 10.1002/ijc.35330

**Published:** 2025-01-24

**Authors:** Joyce Vriend, Ruud Delwel, Dorien Pastoors

**Affiliations:** ^1^ Department of Hematology Erasmus MC Cancer Institute, University Medical Center Rotterdam Rotterdam The Netherlands; ^2^ Oncode Institute Utrecht The Netherlands

**Keywords:** AML, enhancer hijacking, leukemia, oncogene activation

## Abstract

An aggressive subtype of acute myeloid leukemia (AML) is caused by enhancer hijacking resulting in *MECOM* overexpression. Several chromosomal rearrangements can lead to this: the most common (inv(3)/t(3;3)) results in a hijacked *GATA2* enhancer, and there are several atypical *MECOM* rearrangements involving enhancers from other hematopoietic genes. The set of enhancers which can be hijacked by *MECOM* can also be hijacked by *BCL11B*. Enhancer deregulation is also a driver of oncogenesis in a range of other malignancies. The mechanisms of enhancer deregulation observed in other cancer types, including TAD boundary disruptions and the creation of de novo (super‐) enhancers, may explain overexpression of *MECOM* or other oncogenes in AML without enhancer hijacking upon translocation. Gaining mechanistic insight in both enhancer deregulation and super‐enhancer activity is critical to pave the way for new treatments for AML and other cancers that are the result of enhancer deregulation.

## Graphical abstract

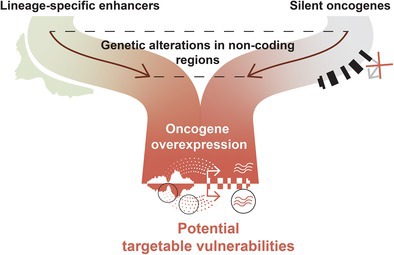

## 1 INTRODUCTION

Acute myeloid leukemia (AML) is the most common type of acute leukemia in adults and is characterized by a clonal expansion of immature myeloid cells. Because of the variety of genetic and epigenetic lesions leading to AML, there is a large heterogeneity of subtypes, which are associated with different prognostic outcomes.^1^

Chromosomal rearrangements in AML frequently result in the fusion of coding sequences, giving rise to fusion transcripts and consequently fusion proteins. These fusion proteins drive oncogenic transformation by repressing the expression of genes required for myeloid development (reviewed in Martens et al.^2^). However, not all chromosomal rearrangements give rise to fusion proteins, as they can also involve noncoding regions including regulatory elements. This has led to the theory that rearrangements involving these regions might result in transcriptional deregulation, which brings about aberrant oncogene expression (reviewed in Bhagwat et al.^3^). Understanding how those regulatory elements drive oncogene expression is pivotal to comprehend the pathology of those AML subtypes. This insight will also help to understand how aberrant gene regulation drives transformation in other hematological and nonhematological cancer types. In this review, we will use aberrant regulation of *MECOM* by ectopic enhancers in AML with chromosome 3q26 rearrangements as a paradigm to discuss mechanisms of aberrant gene control in cancer.

**Gene regulation by enhancers**

Enhancers control developmental‐ and tissue‐specific gene expression patterns (recently reviewed^4^, ^5^, ^6^, ^7^). These regulatory sequences are not protein coding and can be located relatively far away from their target promoters. They contain binding sites for transcription factors (TFs), DNA‐binding proteins which recruit co‐activators such as P300/CBP. These co‐activators modify proteins that are critical for subsequent promoter activation. Some enhancers consist of single elements, while there are also large enhancer clusters, called super‐enhancers that cooperatively regulate target promoters. Long‐distant enhancers often require insulator proteins such as CCCTC‐binding factor (CTCF) for tethering enhancer to the promoter (recently reviewed in Bower and Kvon^8^). These interactions typically occur within boundaries of topologically associated domains (TAD).

## 2 ENHANCER REARRANGEMENTS LEADING TO *MECOM* EXPRESSION IN AML

**FIGURE 1:**
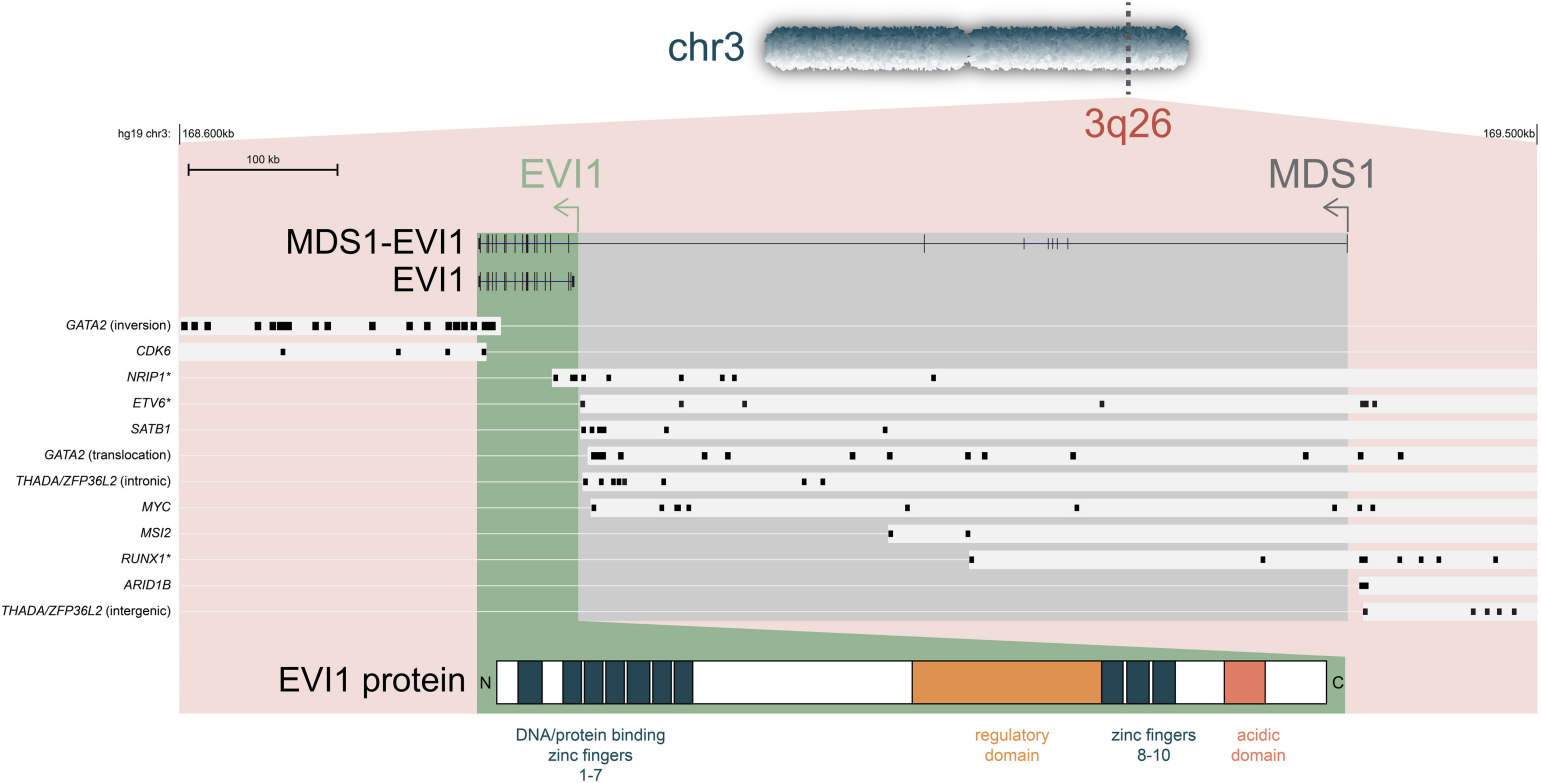
*MECOM* locus with indicated breakpoint locations in MDS/AML. The black boxes denote individual breakpoints of patients. For each breakpoint cluster with the same partner gene, the commonly translated segment is highlighted, which depicts the part of the *MECOM* locus that is now replaced by a novel enhancer. The exons of *MDS1* and *EVI1* are colored in grey and green, respectively. * denotes translocation leading to fusion transcript/protein.

**FIGURE 2:**
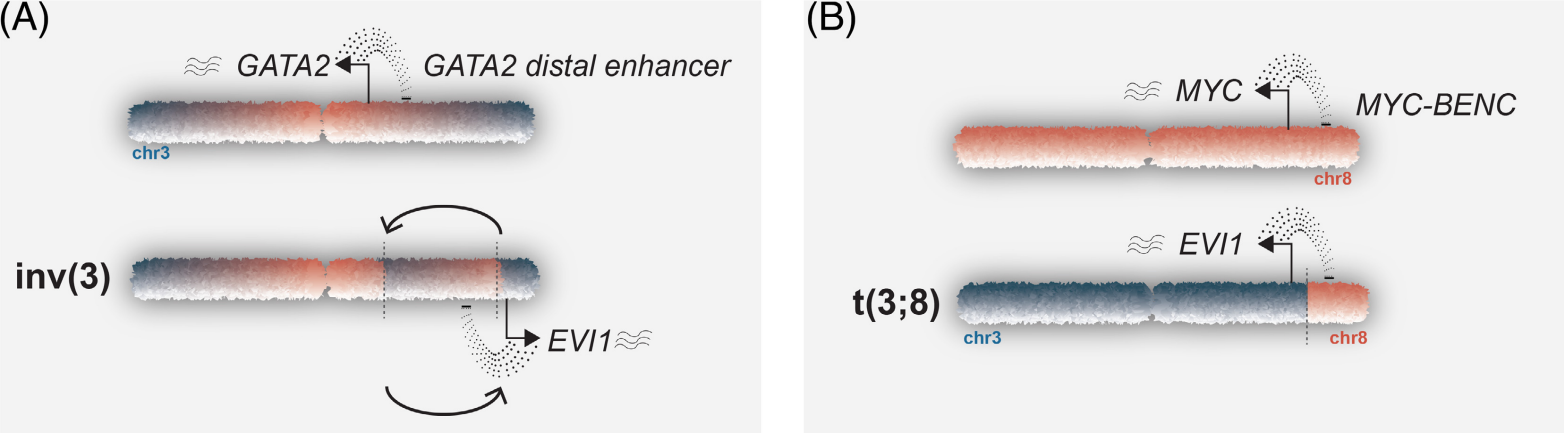
Illustration of two chromosomal abnormalities leading to EVI1 overexpression. (A) In inv(3) patients, an inversion between 3q21 and 3q26 leads to activation of *EVI1* by a distal enhancer of *GATA2*. (B) In t(3;8), the q‐arms of chromosome 3 and 8 are swapped, leading to activation of *EVI1* by a distal *MYC* enhancer.

Enhancer rearrangements were first identified in patients with lymphoid malignancies. In these cells, either the immunoglobulin (Ig)‐ or T‐cell receptor (TCR) genes translocate to oncogenes and drive transcription (reviewed in previous studies^5^, ^7^). The Ig and TCR regions are subject to RAG‐recombination at multiple stages in development and these translocations are the result of errors by these recombination events in those cells. Translocations of enhancers, however, can also occur in other cancer types that are not subject to RAG recombination. In AML, recurrent enhancer translocations affect the *MECOM* locus at chr3q26. *MECOM* encodes important proteins involved in hematopoietic stem cell maintenance in the bone marrow. This is a relatively large locus, encoding for a long isoform, containing *MDS1* and *EVI1* exons, and a short isoform, containing only *EVI1* exons (Figure 1). In AML with *MECOM* rearrangements, the short form *EVI1* and not the long form *MDS1‐EVI1* is overexpressed.^9^, ^10^, ^11^ The two most common abnormalities driving *EVI1* expression in AML are inv(3)(q21;q26.2) or t(3;3)(q21;q26.2) (Figure 2A). In both situations, a long‐ distant enhancer of *GATA2* (3q21) is repositioned near *MECOM* (3q26).^10^, ^12^

**FIGURE 3:**
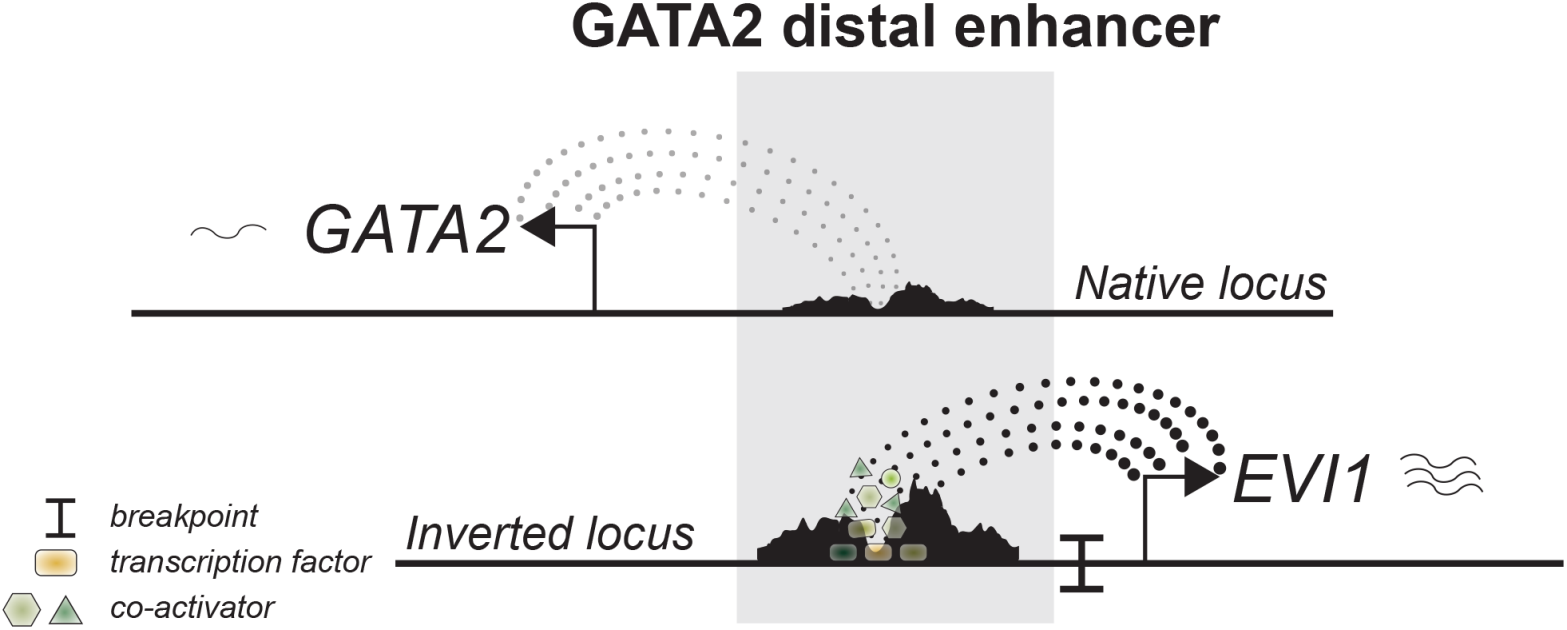
*GATA2* enhancer hijacking leads to overactivation of distal *GATA2* enhancer which is evident by skewed marker occupancy by ChIP‐seq.

This alteration enables the promoter of *EVI1* to interact with the translocated enhancer of the *GATA2* gene and drive *EVI1* overexpression (Figure 3). The enhancer does not activate the promoter located 5′ of the *MDS1* exons and therefore *MDS1‐EVI1* is not expressed in those leukemias. The translocation of the GATA2 enhancer causes two simultaneous events: monoallelic *EVI1* expression from the translocated allele, and heavily skewed *GATA2* expression from the nontranslocated allele. *GATA2* transcription from the rearranged allele is severely reduced due to the loss of the enhancer that was donated to *MECOM*.^10^, ^13^ *GATA2* haploinsufficiency is indeed able to accelerate leukemia development in mouse models of t(3;3)/inv(3).^14^ While this observation clearly shows that the enhancer is also active in its native locus, it becomes much more active when it resides in its new locus, leading to heavily skewed occupancy of many factors to the rearranged enhancer (Figure 3). As a result, *MECOM* is more vulnerable than *GATA2* to disruptions in this enhancer. For example, a MYB motif in the hijacked enhancer is critical for *EVI1* transcription but dispensable for *GATA2* expression.^15^ Thus, the activity of an enhancer is not only determined by its intrinsic capacities, but also by its genomic location.

**FIGURE 4:**
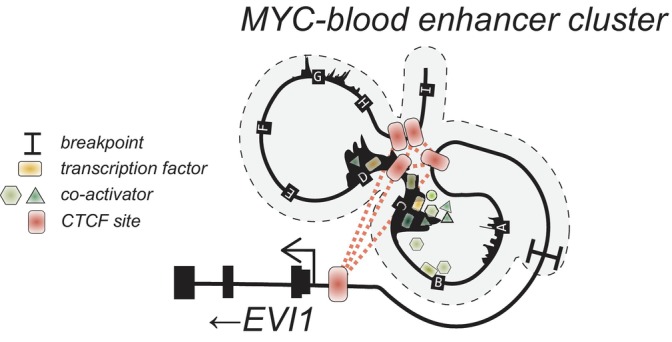
Diagram illustrating interaction of *MYC* with the promoter of *EVI1* in *MECOM*.

*EVI1* overexpression is not restricted to AMLs with inv(3)/t(3;3). Another recurrent 3q26 rearrangement in AML is translocation t(3;8)(q26;q24), in which a *MYC* super‐enhancer (8q24) relocates toward *MECOM* (Figure 2B). In t(3;8) AMLs, an already existing *MYC* super‐enhancer, called Blood ENhancer Cluster (BENC) drives *EVI1* overexpression.^16^ BENC consists of multiple enhancer modules that work in a combinatorial and additive manner.^16^, ^17^ It extends over a region of 150 kb and is located at 1.7 Mb downstream of the *MYC* gene at the nonrearranged allele. BENC harbors at least nine potential activation modules of approximately 1 kb in size each, of which one, module C, is critical for *EVI1* activation in t(3;8) AML.^16^, ^17^ Multiple CTCF binding sites in BENC and one near the *EVI1* promoter are also critical for super‐enhancer driven *EVI1* transcription.^16^ Those sites are essential for promoter‐to‐enhancer looping, allowing enhancement of transcription driven by TFs binding to the enhancers (Figure 4). Thus, CTCF‐looping as well as TF‐driven activation are both essential for oncogene transcription by hijacked enhancers in AML.

## 3 *EVI1* HIJACKS OTHER ENHANCERS IN AML WITH ATYPICAL REARRANGEMENTS

In addition to *MYC‐BENC* and *GATA2* distal enhancer, several other translocations involving *MECOM* have been reported.^11^, ^18^ Other recurrent (but rare) translocations include *THADA/ZFP36L2* for t(2:3)(p21:q26), *CDK6* for t(3:7)(q26:q21), and *ARID1B* for t(3:6)(q26:q21) were reported by Ottema and colleagues.^11^ In addition, they proposed additional candidate partner genes had donated enhancers in rare rearrangements^11^ (Table 1). Most breakpoints have in common that they separate a target promoter from a HEPTAD‐binding, active enhancer cluster in hematopoietic progenitors (FLI1, ERG, GATA2, RUNX1, TAL1, LYL1, LMO2).^27^ CTCF sites in those hijacked super‐enhancers may be essential for promoter interaction, as in *MYC‐BENC* driven *EVI1* overexpression (Figure 4). To mechanistically determine whether and how each of those enhancers drive *EVI1* transcription requires the generation of sophisticated enhancer/*MECOM* models as previously reported for *MYC/MECOM*.^16^

**TABLE 1:** Putative enhancer elements activating EVI1.

	Partner arm	Coordinate likely core (hg38)	Original target	Enhancer name	Recurrent	Other genes
Intergenic	3p24.3	chr3: 18,208,875–18,209,276	SATB1		Y	BCL11B^19^
	8q24.21	chr8: 129,582,024–129,582,425	MYC	BENC element C	Y	BCL11B^19^
	7q21.2	chr7: 92,755,125–92,755,526	CDK6		Y	BCL11B,^19^ MNX1^20^
	17q22	chr17: 57,438,154–57,438,555	MSI2		Y	
	2p21	chr2: 42,949,150–42,949,551	THADA/ZFP36L2		Y	PRDM16^21^
	3q21	chr3: 128,603,426–128,604,425	GATA2	‐117 kb	Y	PRDM16^22^
Intronic^a^	12q22.12	chr21: 35,043,128–35,052,924	RUNX1	*RUNX1* Distal Promoter	Y	BCL11B^19^
		chr21: 35,017,769–35,036,211		*RUNX1* p22 Intronic enhancer	Y	
		chr21: 34,877,719–34,901,925		*RUNX1* p161 promoter	Y	
	12p13.2	chr12: 11,704,802–11,791,354	ETV6	*ETV6* intronic enhancer	Y	BCL11B,^19^ CDX2,^23^ MN1,^24^ IL3,^25^ MNX1^26^
	21q21.1	chr21: 15,050,535–15,072,908	NRIP1	*NRIP1* promoter	Y	
		chr21: 15,133,144–15,137,286		*NRIP1* upstream enhancer	Y	
Nonrecurrent^b^	3p23	chr3: 31,223,039‐31,230,622	STT3B		N	
	7p22.1	chr7: 5,456,955–5,458,911	TNRC18		N	
	3q25.33	chr3: 160,170,419–160,182,035	IL12A‐AS1		N	
	6q21	chr6: 109,292,077–109,310,663	CD164		N	
	10q21.2	chr10: 61,755,364–61,771,720	ARID5B		N	

^a^ Intronic breakpoints lead to fusion transcripts/proteins as well as enhancer/promoter hijacking.

^b^ BPs with no clear enhancer and interaction: *FAM153B, TNKS, PROM1, GTF2E1 JARID2, DMTF1*.

Some recurrent *MECOM* translocations lead to the generation of fusion genes and proteins, such as *RUNX1‐MECOM* or *ETV6‐MECOM* in translocation t(3;21)(q26.2;q22) and t(3;12)(q26.2;p13.2), respectively.^11^, ^18^, ^28^ These fusion proteins include the promoter and upstream regulatory elements of the partner genes.^29^, ^30^ Similarly, as in enhancer hijacked *EVI1* overexpressing AMLs, HEPTAD binding sites, which mark predicted active enhancers, are present near the *RUNX1‐EVI1* and *ETV6‐EVI1* fusion genes in t(3;21)(q26.2;q22) and in t(3;12)(q26.2;p13.2) AML cells. We hypothesize that in each of the different 3q26 rearrangements, *EVI1* overexpression is driven by an enhancer that has been donated by genes that are normally expressed in the same “target cells” within the HSPC pool in the bone marrow.

## 4 A COMMON SET OF ENHANCERS IS RECURRENTLY HIJACKED BY OTHER ONCOGENES IN LEUKEMIA

*EVI1* is not the only gene in leukemia which can be overexpressed by the hijacked enhancers reported in Table 1. The *GATA2* enhancer may be hijacked by *PRDM16*, a gene that is highly homologous to *EVI1*, in AML with a t(1;3)(p36.3;q21.1) rearrangement. This translocation involves the GATA2/*RPN1* locus, suggesting that the *GATA2* enhancer drives *PRDM16* overexpression in t(1;3)(p36.3;q21.1) AML.^22^ In addition, a *THADA/ZFP36L2* translocation has been reported for *PRDM16* in AML.^21^ Several of the enhancers hijacked by *EVI1* are also recurrently repositioned near the *BCL11B* gene driving acute leukemias of ambiguous lineages (ALAL).^19^ *BCL11B* becomes overexpressed through rearrangements that juxtaposes it next to super‐enhancers of *ARID1B*, *MYC*, *CDK6*, *ETV6*.^19^ Conversely, a BCL11B enhancer can also activate *TLX3* in pediatric leukemia.^25^ In AML with a translocation t(12;13)(p13;q12) an *ETV6‐CDX2* fusion gene and protein is generated.^23^ As with *ETV6‐EVI1*, the *CDX2* gene is fused to a small 5′ part of *ETV6*. This fusion gene comes under the control of the promoter and enhancers of *ETV6*.^23^ Intronic enhancers of *ETV6* can also activate expression of IL‐3 in ALL through t(5;12)(q31;p13).^31^ In AML, it can also activate *MN1* through t(12;22)(p13.2;q12.1). The latter translocation leads to breakpoints 5′ of *ETV6*, while breakpoints with *IL3*, *MECOM*, *BCL11B*, and *CDX2* are intronic or 3′. Despite the inverse breakpoint orientation with respect to the gene, it leads to interaction of the *MN1* promoter with the *ETV6* intronic enhancer.^24^ In addition, *CDK6* locus is involved in *MNX1* overexpression.^20^ In those leukemias, *MNX1* which is positioned at the very tip of the q‐arm of chromosome 7, is positioned next to the enhancer of the *CDK6*‐locus (more centromeric at 7q), as the result of a large 7q deletion.^20^, ^26^ Thus, it appears that in both AML and ALAL, the enhancers of the same partner genes are of importance for the activation of different oncogenes. An overview of the distinct enhancers that are found to be repositioned due to chromosomal rearrangements are provided in Table 1. Thus, multiple oncogenes can hijack a unique set of enhancers, making the regulators of those enhancers as important as the oncogene for transformation.

## 5 ENHANCER HIJACKING IN NONHEMATOLOGICAL CANCERS

Enhancer rearrangements may also drive oncogene overexpression in other cancer types. In neuroblastoma, researchers observed that several super‐enhancers can translocate proximal to the *TERT* gene, which encodes for telomerase reverse transcriptase, causing *TERT* overexpression.^32^ In medulloblastoma, the *GFI1* and *GFI1B* oncogenes become juxtaposed to multiple super‐enhancer regions.^33^ In adenoid cystic carcinoma, *NFIB* and *TGFBR3* loci, which contain super‐enhancers, are translocated up‐ or downstream of the *MYB* gene.^34^ Interestingly, MYB can bind to these translocated super‐enhancers, creating a positive feedback loop which sustains *MYB* overexpression.^34^ In acinic cell carcinoma of the salivary gland, an active enhancer region of the *SCPP* gene cluster is moved towards the *NR4A3* oncogene.^35^ These discoveries show that this translocation‐ and inversion‐based mechanism of enhancer hijacking is used by other oncogenes and can happen in other cancers and is consequently not unique to hematological malignancies. Like in AML, frequency of enhancer hijacking reported in solid tumors is low. It is still unclear whether this low incidence represents the low frequency of its occurrence or whether it is frequently overlooked.

## 6 OTHER MECHANISMS OF ENHANCER DYSREGULATION

EVI1 is activated by unknown mechanisms in a large fraction of EVI1‐expressing patients, of which many also exhibit skewed expression. Other mechanisms of enhancer‐driven oncogene activation may provide a clue about alternative ways EVI1 may become overexpressed.

### 6.1 Duplications and somatic mutations create new enhancers

The overexpression of an oncogene can also be the result of enhancer duplications, or even of the creation of a new enhancer. In human epithelial cancers, several oncogenes, such as *MYC*, *KLF5*, *USP12*, and *PARD6B*, are overexpressed as result of focal copy number amplifications of noncoding regions that harbor super‐enhancers.^36^ In other cancer types, including AML, T‐ALL, and neuroblastoma, *MYC* overexpression is associated with amplification of super‐enhancers downstream of the *MYC* gene^37^, ^38^; reviewed in Shi et al.^39^ These findings suggest that enhancer amplification might be a common mechanism of oncogene activation. *BCL11B* has been reported to be overexpressed in childhood leukemias, due to enhancer amplifications 3′ of the gene.^19^, ^40^ Besides, in primary gastric adenocarcinoma, enhancer amplification causes overexpression of the *CCNE1* oncogene, implying that these mechanisms can be present at the same time.^41^

In T‐ALL, recurrent 2‐ to 18‐bp insertions upstream of the *TAL1* oncogene have been identified which create de novo enhancers.^42^, ^43^ These insertions introduce a binding motif for MYB, resulting in the creation of a super‐enhancer that drives *TAL1* overexpression.

Similarly, the creation of an active enhancer by the introduction of a *MYB* binding motif also drives *LMO1* and *LMO2* overexpression in T‐ALL.^44^, ^45^, ^46^ In the case of *LMO1*, this is the result of a heterozygous C‐to‐T single nucleotide mutation 4 kb upstream of the *LMO1* gene, and in the case of *LMO2*, this is a result of an 8 bp insertion in the *LMO2* locus.^44^, ^45^, ^46^ In contrast to the *TAL1* cases, these new enhancers do not classify as super‐enhancers.^44^, ^45^ In fact, somatic mutations can also create new TF binding sites within a promoter, which has for instance been observed in the *TERT* promoter where it results in an increased *TERT* transcription.^47^

### 6.2 TAD boundary disruption

Another way to enable enhancer hijacking is by disruption of TAD boundaries. TAD boundaries restrict the interactions of regulatory elements and genes within the same TAD. Thus, a disruption of such a boundary can result in interactions between a promoter of an oncogene in one TAD and an otherwise inaccessible enhancer in a neighboring TAD.

#### 6.2.1 Deletions

**FIGURE 5:**
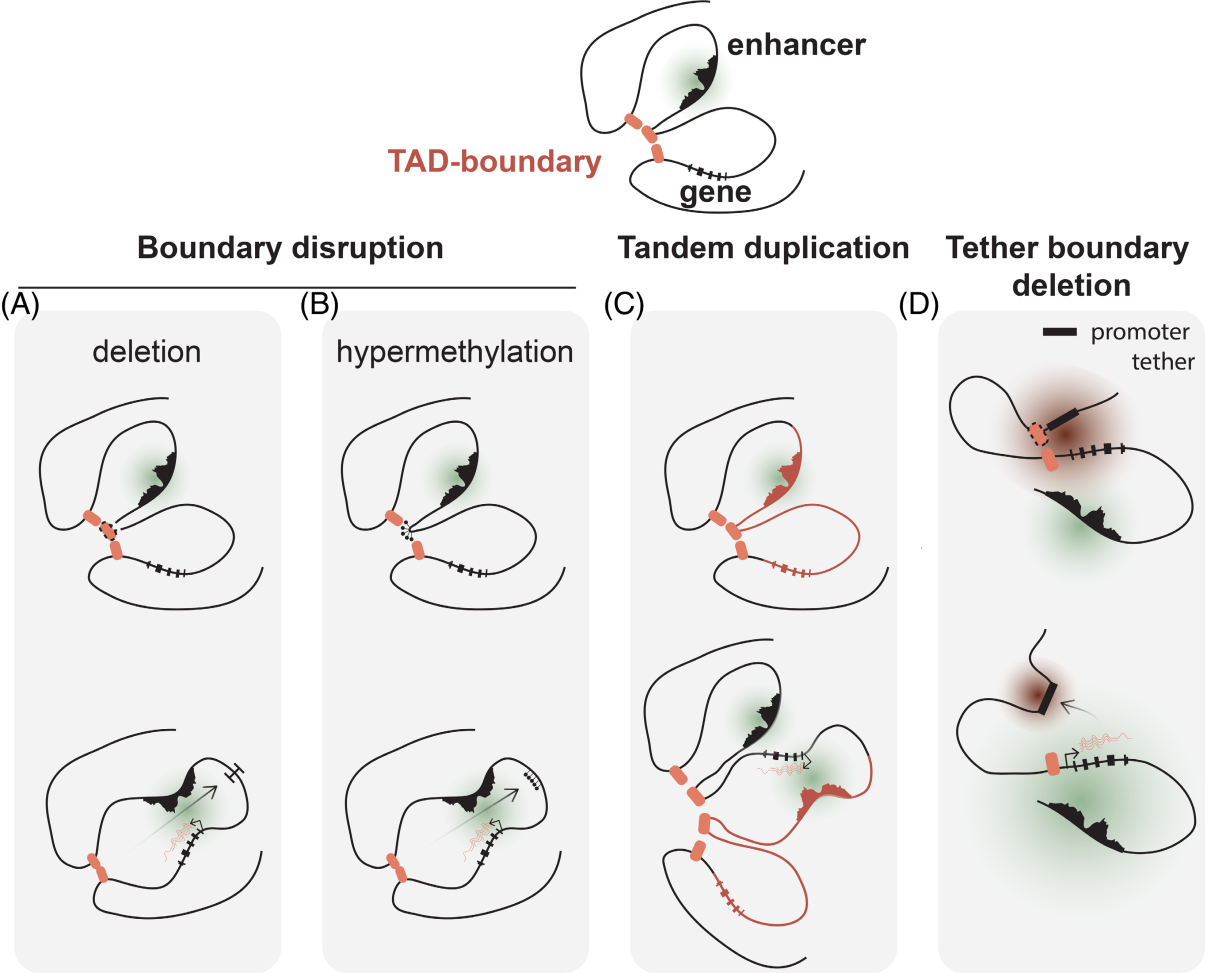
Boundary disruptions or amplifications can enable oncogene activation. Boundary disruption by CTCF deletion (A) or methylation (B), tandem duplication (C) or deletion of a promoter tether CTCF site (D).

A TAD boundary may be disrupted by focal deletions (Figure 5A). In T‐cell acute lymphoblastic leukemia (T‐ALL), a deletion of the boundary between the *TAL1‐* and *STIL* loci disrupts a CTCF site, placing the *TAL1* oncogene under control of an enhancer outside its neighborhood.^48^ A similar mechanism has been observed in high‐hyperdiploid BCP ALL.^49^ In 3.9% of these cases, the *FLT3* gene is located next to an enhancer as a result of a 13q12.2 microdeletion involving a TAD boundary immediately downstream of the *FLT3* promoter.^49^ Although the normal *FLT3* enhancer is deleted, another enhancer distal to the deletion breakpoint that is normally insulated from *FLT3* will now hyperactivate transcription of *FLT3*.^49^ In lung squamous cell carcinoma, recurrent deletions were identified 103 kb downstream of *IRS4*, which overlap with an insulator region.^50^ Loss of the TAD boundary caused the spreading of the active chromatin mark H3K27ac around the deletion.^50^ As a result, stronger interactions between the *IRS4* promoter and its downstream enhancer were established, leading to the overexpression of the oncogenic driver *IRS4*.^50^ Deletions at the same region were also associated with *IRS4* overexpression in other types of cancer, including sarcomas, cervical squamous carcinomas, and benign uterine leiomyomas.^50^ It is debatable whether this last situation can be called enhancer hijacking, as the enhancer already interacts with this gene in the normal situation. Nevertheless, it clearly demonstrates that deletion of a TAD boundary allows enhancers to hyper‐activate transcription of normally “inaccessible” proto‐oncogenes in a unique manner.

#### 6.2.2 Hypermethylation

Another way by which a TAD boundary may be disrupted allowing enhancers to interact with a gene in a distinct TAD is through the hypermethylation of CTCF sites (Figure 5B). The CTCF sites in gliomas with a gain‐of‐function *IDH* mutation are hypermethylated, as the mutant IDH protein interferes with TET enzymes, which normally remove DNA methylation.^51^ As CTCF binding is proven to be methylation‐sensitive, this results in a loss of insulation between TAD domains.^51^ This change may subsequently result in the aberrant interaction between the *PDGFRA* gene and a constitutive enhancer located in a neighboring TAD. Although *IDH* mutations are also quite common in AML, no cases have been observed where this is associated with the activation of the *PDGFRA* oncogene or other oncogenes. It is possible that in IDH mutant AML changes in DNA methylation affect the transcription of other genes and loci, but to our knowledge this has not been reported.

#### 6.2.3 Tandem duplication

A third mechanism of TAD boundary disruption is the positioning of an oncogene directly adjacent to an enhancer through a tandem duplication (Figure 5C). In colorectal cancer, an *IGF2* locus tandem duplication was found to be associated with a strong interaction between the *IGF2* oncogene and a normally inaccessible super‐enhancer.^50^ It appeared that the copy of *IGF2* was placed in head‐to‐tail orientation to this super‐enhancer, overcoming the interference of an insulator. A very similar mechanism also causes *IGF2* overexpression in primary gastric adenocarcinoma.^41^ It was also demonstrated that tandem duplication of a super‐enhancer overlapping *DIRC3* and Hedgehog ligand *IHH* leads to formation of novel interactions between the promoter of *IHH* and the *DIRC3* enhancer in meningioma.^52^

#### 6.2.4 Promoter tether

A fourth type of boundary disruption was recently described where the promoter of T‐ALL oncogene *IRX3* has simultaneous CTCF‐dependent interactions with a repressive element in an intron of neighboring gene *FTO*, as well as a super‐enhancer located in *CRNDE*. When the repressive *FTO* element is interacting with the *IRX3* promoter through a CTCF binding, transcription is repressed. In T‐ALL, focal deletions of the CTCF site adjacent to the repressive element releases its promoter tether and this results in exclusive interaction of the *IRX3* promoter with the super‐enhancer, leading to mono‐allelic overexpression of *IRX3* (Figure 5D).^53^

These previously described mechanisms are not the only ways by which enhancer hijacking via a TAD disruption may occur. For instance, a TAD fusion event enables the *MYC* promoter to interact with a NOTCH‐bound distal super‐enhancer in T‐ALL samples as result of CTCF binding changes.^54^ However, these changes are not caused by genomic mutations or hypermethylation, but by a reduced chromosome accessibility of unknown origin. The resulting TAD boundary disruption will have the same effect as depicted in Figure 5A,B.^54^

The previous findings show that enhancer‐driven oncogene activation occurs in different cancer types and can be driven by several different mechanisms. These mechanisms were discovered by focusing on the mutations and structural variations in the noncoding regulatory regions. Some of these mechanisms could perhaps underlie the cases of EVI1 overexpression in AML that cannot be explained by translocation‐ or inversion‐based enhancer hijacking. Besides, it could be that in AML those distinct mechanisms of oncogene activations and new promoter to enhancer interactions occur much more often but are missed simply because we have not yet extensively enough developed and applied high‐throughput methods to determine such defects in cancer and leukemia in particular using large patient cohorts.

## 7 CONCLUDING REMARKS AND PERSPECTIVES

### 7.1 Hallmarks of enhancer misregulation

In this review, focusing on *MECOM* misregulation in AML, we have touched upon several hallmarks of enhancer‐based oncogene activation. Firstly, enhancer dysregulation events often involve genetic alternations, which can differ in magnitude from chromosomal rearrangements to focal deletions and somatic mutations. Enhancers that are hijacked in cancer appear to be strong lineage‐specific enhancers in the cell of origin and are often super‐enhancers. Known enhancer dysregulation events are often detected using skewed gene expression, which is a powerful tool for detecting heterozygous abnormalities. Despite enhancer hijacking events being strong cancer drivers, they can also create a vulnerability. For example, enhancer hijacking can change dependency of the target gene on gene insulation: frequently, hijacking is accompanied by abnormal use or dependency on certain CTCF binding sites. In addition, a hijacked enhancer may have differential factor dependencies in its native context compared to the hijacked state. This opens an opportunity for pharmacological targeting of these enhancers.

### 7.2 Computational approaches to detect enhancer hijacking

Recently, several computational approaches have been developed to detect novel enhancer hijacking events in whole‐genome‐ and transcriptome sequencing data. HYENA,^55^ CESAM,^50^ and SVExpress^56^ correlate gene expression with breakpoint locations, enabling identification of recurrent translocations. Cis‐X^57^ allows for detecting single‐sample events by employing monoallelic expression with matched control. PyJacker^20^ can detect single events without needing a matched control. Enhancer hijacking can also be detected using HiChIP‐data (HAPI^58^) or Hi‐C (NeoLoopFinder^59^). These analyses applied on patient cohorts are essential for discovery of novel enhancer/promoter hijacking events.

### 7.3 Challenges

A key challenge in identifying noncoding drivers is the amount of noncoding information in the genome. Current approaches frequently use SNPs to detect heterozygous expression. This is a powerful tool but limited to genes containing SNPs in the samples of interest. In addition, there might be driver events that are homozygous and therefore not detected in this way. Studying enhancers and genes separated by long distances using SNPs has the added challenge of linking allelic variants. A key challenge is that with a large variety of mechanisms of gene dysregulation by enhancers, there are only a few patient samples for each abnormality, which are therefore more difficult to detect.

### 7.4 Future directions

The finding that both *BCL11B* and *MECOM* can be hijacked by the same set of HSPC‐specific enhancers suggests there might be other oncogenes in hematopoiesis that are hijacked by a similar mechanism. This makes hijacked enhancers equally important as their activated oncogenic targets. We therefore propose deep sequencing experiments in AML capturing the set of identified enhancers and search for breakpoints and new partner genes that can be controlled by those hijacked enhancers. Aside from skewed expression, skewed factor occupancy or ATAC signal could also be considered when exploring novel enhancer hijacking events. Another approach could be to do this with Micro‐capture C using the enhancers as bait.^60^ Changed factor dependency between hijacked enhancer versus their nonhijacked counterpart are very interesting from a gene regulatory point of view. Apart from investigating these dependencies for pharmacological intervention, it is also interesting to explore how this dependency is established. Perhaps there are repressive mechanisms at play in the native locus that are not joining the translocation. Engineering cellular models to study enhancers in their native or hijacked contexts is possible using CRISPR‐Cas9 technology and a key way forward to understand this type of driver events in cancer in the appropriate cellular context. Massive parallel reporter assays, potentially combined with synthetic enhancer design could be employed to define the requirements of enhancer translocations and answer important biological questions regarding enhancer biology.^61^, ^62^

## AUTHOR CONTRIBUTIONS

**Joyce Vriend:** Writing – original draft; conceptualization. **Ruud Delwel:** Writing – review and editing; funding acquisition; conceptualization; supervision. **Dorien Pastoors:** Writing – review and editing; visualization; conceptualization; supervision.

## CONFLICT OF INTEREST STATEMENT

The authors declare no conflicts of interest.
